# A partial proteome reference map of the wine lactic acid bacterium *Oenococcus oeni* ATCC BAA-1163

**DOI:** 10.1098/rsob.130154

**Published:** 2014-02-26

**Authors:** María de la Luz Mohedano, Pasquale Russo, Vivian de los Ríos, Vittorio Capozzi, Pilar Fernández de Palencia, Giuseppe Spano, Paloma López

**Affiliations:** 1 Departamento de Microbiología Molecular y Biología de las Infecciones, Centro de Investigaciones Biológicas, Consejo Superior de Investigaciones Científicas, Calle Ramiro de Maeztu 9, Madrid 28040, Spain; 2 Dipartimento di Scienze Agrarie, degli Alimenti e dell'Ambiente, University of Foggia, Via Napoli 25, Foggia 71122, Italy

**Keywords:** *Oenococcus oeni*, proteome, two-dimensional electrophoresis

## Abstract

*Oenococcus oeni* is the main lactic acid bacterium that carries out the malolactic fermentation in virtually all red wines and in some white and sparkling wines. *Oenococcus oeni* possesses an array of metabolic activities that can modify the taste and aromatic properties of wine. There is, therefore, industrial interest in the proteins involved in these metabolic pathways and related transport systems of this bacterium. In this work, we report the characterization of the *O. oeni* ATCC BAA-1163 proteome. Total and membrane protein preparations from *O. oeni* were standardized and analysed by two-dimensional gel electrophoresis. Using tandem mass spectrometry, we identified 224 different spots corresponding to 152 unique proteins, which have been classified by their putative function and subjected to bioinformatics analysis.

## 2. Introduction

*Oenococcus oeni* is the most important lactic acid bacterium (LAB) in the wine industry, because it de-acidifies the wine following the alcoholic fermentation, a process termed malolactic fermentation. Nevertheless, the harsh wine environment represents a challenge to the survival of *O. oeni* and can strongly affect the successful outcome of the vinification [1]. Therefore, a better understanding of the molecular mechanisms related to the stress adaptation and technical performance of *O. oeni* is crucial for the characterization and selection of strains for industrial purposes [2,3].

Currently, a fully complete genome sequence is available only for the *O. oeni* PSU-1 strain [4]. However, the genome sequences of ATCC BAA-1163 and AWRIB129, AWRIB202, AWRIB304, AWRIB318, AWRIB418, AWRIB419, AWRIB422, AWRIB429, AWRIB548, AWRIB553, AWRIB568, AWRIB576 are in assembly [5].

Unluckily, a recombinant approach in *O. oeni* usually does not give satisfactory results. Despite many attempts over the years, only in rare cases has it been possible to insert foreign genetic material in *O. oeni* [6–9]. Therefore, the main strategy for molecular analysis of *O. oeni* has been based on the heterologous expression of genes or clusters of interest. In particular, the malic acid metabolism has been extensively investigated as well as the production of compounds affecting wine quality or safety such as flavours or biogenic amine formation (for a comprehensive review, see Bartowsky [10]). The adaptive stress response of *O. oeni* in wine uses principally three mechanisms: (i) the establishment of a proton-motive force generated by the malolactic fermentation [11], (ii) the synthesis of heat-shock proteins [12] and (iii) physico-chemical changes in the membrane composition [13,14].

Although the significance of these mechanisms is clear, few authors have addressed the study of *O. oeni* from a proteomic perspective. Two-dimensional gel analysis of total cellular proteins provides a global overview on the real biological response under specific conditions. Currently, only few comparative analyses of *O. oeni* proteomes have been reported [15–17]. Detection of 81 out of 186 differently expressed peptides was observed during starvation conditions, although none of the differently expressed spots were identified [16]. Cecconi *et al*. [17] were able to obtain high-resolution two-dimensional gel maps of *O. oeni*, when investigating the acclimation of freeze-dried cultures. The same authors attributed the changes in two-dimensional gel profiles mainly to a set of 20 cytosolic proteins involved in stress response and metabolic processes. None of these studies provided information on the membrane proteome.

In this work, we report the characterization of the membrane and cytosolic proteomes of *O. oeni* ATCC BAA-1163 [18]. In addition, we describe a standardized and optimized method to obtain membrane protein extracts from *O. oeni*. In the course of this study, 224 different spots corresponding to 152 different proteins of the *O. oeni* ATCC BAA-1163 proteome have been identified, classified by their putative function and subjected to bioinformatics analysis. This partial proteomic approach has allowed us to draw a proteome reference map of *O. oeni*, which could help subsequent comparative analysis as well as representing a valuable source of information for the validation of annotated genes.

## 3. Material and methods

### 3.1. Bacterial strain and growth conditions

*Oenococcus oeni* ATCC BAA-1163 (formerly *O. oeni* IOB 8413, [18]) was grown at 30°C in FT80 broth [19] at pH 5.3, under anaerobic conditions (AnaeroGen 3.5 l, Oxoid, Basingstoke, Hampshire, UK). A draft version of *O. oeni* ATCC BAA-1163 genome is available under the GenBank accession number AAUV00000000.1. This draft has a GC content of 37.9% and it contains a total of 1398 predicted genes and 280 pseudogenes.

### 3.2. Preparation of protein extracts

Stock culture of *O. oeni* ATCC BAA-1163 (stored at −80°C) was diluted 1 : 1000 in 1800 ml of fresh medium. When the culture reached the end of the exponential phase (OD_620_ = 1.2), cells were used to prepare both total and membrane protein extracts. Bacteria were sedimented by centrifugation (11 000×*g* for 20 min at 4°C) and washed with 200 ml of cold 0.1 M potassium phosphate buffer (0.1 M KH_2_PO_4_, 0.1 M K_2_HPO_4_, Merck, Darmstadt, Germany) pH 6.0. The cell pellet then was frozen at −80°C. Experiments were performed in triplicate.

### 3.3. Preparation of total extracts

The frozen pellet was defrosted and resuspended in 60 ml of 0.1 M potassium phosphate buffer supplemented with 30 µg ml^−1^ of protease-free DNase I (Roche Diagnostics GmbH, Mannheim, Germany), 10 mM MgSO_4_ (Merck) and 1× concentrate Complete Protease Inhibitor cocktail (Roche Diagnostics GmbH). Total extracts were obtained by passing the cells four times through a French Press at 12 000 lb in^−2^. Cell debris was removed by centrifugation (1252×*g*, 15 min, 4°C). The supernatant was designated total protein extracts and frozen at −80°C.

### 3.4. Preparation of membrane extracts

Thirty millilitres of total protein extracts were diluted with 120 ml of 0.1 M sodium carbonate (Merck) pH 11.0, in order to optimize the linearization of membrane vesicles. This solution was gently shaken at 0°C for 1 h then centrifuged at 154 980×*g*, 40 min at 4°C in a Beckman L8-60M Ultracentrifuge (Type 90 Ti rotor). The pellet was resuspended in 10 ml of 50 mM Tris/HCl (Merck) pH 7.3, 1 mM MgCl_2_ (Merck). After another sedimentation by ultracentrifugation (154 980×*g*, 40 min at 4°C), the membranes were resuspended in 2 ml of 50 mM ammonium bicarbonate (Merck). To increase the proportion of membrane proteins, the membrane extracts were supplemented with 13.3 ml of 2 : 1 v/v trifluoroethanol (Sigma-Aldrich, St. Louis, MO, USA)/chloroform (Merck) and stored on ice for 1 h as reported by Pessione *et al*. [20]. After centrifugation (10 000×*g*, 5 min at 4°C), the upper phase was recovered and dried using a vacuum centrifuge. To increase the solubilization of membrane proteins, the samples were treated with the zwitterionic detergent amidosulfobetaine-14 (ASB-14) (Calbiochem, Darmstadt, Germany) [21] and tributyl phosphine (TBP) (Bio-Rad Laboratories, Hercules, CA, USA) by resuspension in the rehydration buffer A (7 M urea (Merck), 2 M thiourea (GE Healthcare, Piscataway, NJ, USA), 1% ASB-14, 40 mM Tris, 2 mM TBP). The samples then were submitted to three cycles of sonication for 30 s and cooling on ice for 30 s. Finally, they were aliquoted and frozen at −80°C.

### 3.5. Protein extraction and determination

The protein preparations were solubilized for 1 h at room temperature with agitation. The total protein concentration present in the extracts was determined by two methods: RC DC Protein Assay (Bio-Rad Laboratories) Kit and Quant-iT technology by the use of the Qubit quantification platform (Invitrogen, Paisley, UK).

### 3.6. Two-dimensional electrophoresis analysis

Triplicate two-dimensional gels for each sample were carried out as described below: 50 μl aliquots of extracts containing 150 µg of proteins were mixed with a buffer containing 7 M urea, 2 M thiourea, 4% CHAPS, 20 mM dithiothreitol, 1% carrier ampholytes pH 3–11 (GE Healthcare), up to a final volume of 100 µl, and applied by Cup Loading to 18 cm IPG strips pH 3–11 NL (GE Healthcare) previously rehydrated with 340 µl of the isoelectrofocusing (IEF) buffer (7 M urea, 2 M thiourea, 4% CHAPS, 0.5% carrier ampholites pH 3–11, 1.2% DeStreak (GE Healthcare)). The first dimension was run at 0.05 mA/IPG strip in the IPGphor IEF System (GE Healthcare) with voltage increase in five steps: 300 V h^−1^ for 3 h, linear gradient up to 1000 V in 6 h, linear gradient up to 8000 V in 3 h and 8000 V h^−1^ until 42000 V h^−1^ was reached. After IEF separation, the strips were equilibrated twice for 10 min in 50 mM Tris–HCl (pH 8.8), 6 M urea, 30% glycerol (Merck), 2% sodium dodecyl sulfate (SDS; Merck) and trace amounts of bromophenol blue (Sigma-Aldrich). The first equilibration solution contained 1% dithiothreitol (Bio-Rad Laboratories), whereas the second contained 4% iodoacetamide (Sigma-Aldrich). The second dimension (SDS-PAGE) was performed on 12.5% polyacrylamide gels (1 mm, 16 × 15 cm). Gels were run at 7 mA per gel overnight maintaining buffer temperature at 4°C. Staining was carried out with SYPRO Ruby Protein Gel Stain from Invitrogen as follows: gel was fixed in 10% methanol, 7% acetic acid (Merck) for 30 min, incubated overnight in SYPRO Ruby staining solution, washed twice with 10% methanol, 7% acetic acid for 30 min, and finally washed twice with water for 10 min. Gels were then scanned in a Typhoon 9400 Variable Mode Imager (GE Healthcare) equipped with a 532 nm excitation laser with the emission filter adjusted to 610 nm and 100 µm resolution. The photomultiplier tube settings were modified to optimize sensitivity to background ratios.

The protein spots present in the two-dimensional gels were matched and quantified with the DeCyder v. 7.0 software (GE Healthcare). For this quantification, stained spots were matched in all gels and used for normalization of the average intensity. The selected spots were automatically excised with the Spot Picker (GE Healthcare).

### 3.7. In-gel protein digestion and sample preparation

Two hundred and twenty-four spots of interest from Sypro Ruby-stained two-dimensional gels were excised manually, deposited in 96-well plates and processed automatically in a Proteineer DP (Bruker Daltonics, Bremen, Germany). The digestion protocol used was based on that of Shevchenko *et al*. [22] with minor variations: gel plugs were washed first with 50 mM ammonium bicarbonate (Sigma-Aldrich) and then with acetronile (ACN) (Scharlau, Barcelona, Spain) prior to reduction with 10 mM dithiothreitol in 25 mM ammonium bicarbonate solution, and alkylation was carried out with 55 mM iodoacetamine in 50 mM ammonium bicarbonate solution. Gel slices were then rinsed first with 50 mM ammonium bicarbonate and then with ACN, and finally were dried under a stream of nitrogen. Modified porcine trypsin (sequencing grade; Promega, Madison, WI, USA) at a final concentration of 16 ng μl^−1^ in 25% ACN, 50 mM ammonium bicarbonate solution was added and the digestion took place at 37 °C for 6 h. The reaction was stopped by adding 0.5% trifluoroacetic acid (TFA) (Sigma-Aldrich) for peptide extraction. The tryptic-eluted peptides were dried by speed-vacuum centrifugation and were resuspended in 4 µl of 33% ACN, 16% isopropanol, 0.5% TFA (MALDI solution). A 0.8 µl aliquot of each peptide mixture was deposited onto a 389-well OptiTOF Plate (Applied Biosystems, Framingham, MA, USA) and allowed to dry at room temperature. A 0.8 µl aliquot of matrix solution (3 mg ml^−1^ of α-cyano-4-hydroxycinnamic acid (Bruker Daltonik) in MALDI solution was then deposited onto dried digest and allowed to dry at room temperature.

### 3.8. MALDI peptide mass fingerprinting, MS/MS analysis and database searching

For MALDI-TOF/TOF analysis, samples were automatically acquired in an ABi 4800 MALDI-TOF/TOF mass spectrometer (Applied Biosystems) in positive ion reflector mode (the ion acceleration voltage was 25 kV to MS acquisition and 1 kV to MSMS) and the obtained spectra were stored into the ABi 4000 Series Explorer Spot Set Manager. PMF and MSMS fragment ion spectra were smoothed and corrected to zero baseline using routines embedded in ABi 4000 Series Explorer software v. 3.6. Each PMF spectrum was internally calibrated with the mass signals of trypsin autolysis ions to reach a typical mass measurement accuracy of less than 25 ppm. Known trypsin and keratin mass signals as well as potential sodium and potassium adducts (+21 and +39 Da) were removed from the peak list. To submit the combined PMF and MS/MS data to Mascot software v. 2.1 (Matrix Science, London, UK), GPS Explorer v. 4.9 was used, searching in the non-redundant NCBI protein database. The mass tolerance for precursors was set to ±50 ppm and to ±0.3 Da for MS/MS fragment ions. Peptide identifications were accepted when scored at greater than 95.0% probability by the Mascot algorithm [23].

## 4. Results and discussion

### 4.1. Protein extraction and protein identification

**Figure 1.**
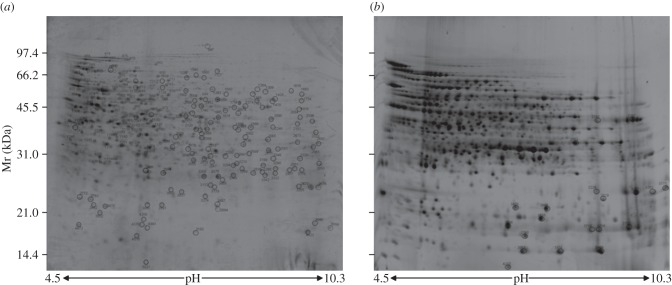
Reference two-dimensional gels from protein preparations. Total protein (*a*) and membrane (*b*) fractions obtained from extract of *O. oeni* ATCC BAA-1163 were analysed in two-dimensional gels, by the use of a nonlinear pH gradient (pH 3.0–11.0) and the second dimension ranging from 150 to 10 kDa.

In order to obtain a reference map representative of both cytoplasmic and membrane proteins, two protein fractions were prepared from three independent *O. oeni* ATCC BAA-1163 cultures for further fractionation by two-dimensional electrophoresis. Thus, after disruption of the cells, we generated a total fraction containing both cytoplasmic and membrane proteins. Membrane proteins are generally poorly represented on two-dimensional gels owing to their low abundance and poor solubility, and to self-aggregation during extraction or fractionation [24]. Recently, Choi *et al*. [25] reported that a sodium carbonate precipitation coupled with ultracentrifugation is an effective method to increase the proportion of cytoplasmic membrane proteins in extracts of *Streptococcus pneumoniae*. We therefore optimized a similar protocol to generate the membrane preparation. In addition, to improve the solubility of proteins, we subjected the sodium carbonate precipitated samples to a delipidation process by treatment with trifluoroethanol/chloroform, as previously described [20,26], and to membrane solubilization by treatment with the zwitterionic detergent ASB-14 [21]. Finally, the membrane extracts were sonicated to increase protein solubility prior to analysis. The two protein preparations were analysed by two-dimensional electrophoresis, as described above, which detected focused polypeptides in the range of pI 4.5–10.3. The second dimension separated in the range from 150 to 10 kDa. Image analysis of representative two-dimensional gels from total and membrane preparations prepared from the same extract of *O. oeni* ATCC BAA-1163 revealed a high degree of overlap of the spots, although the relative proportions of them were different (figure 1). Therefore, we chose the total protein fractions analysis for the identification of 203 spots, which are depicted in figure 1*a* (gel A). An additional 21 spots, mainly characterized by alkaline isoelectric point (pI) and low molecular weight (MW), were detected on two-dimensional gel analysis of the membrane fraction (figure 1*b*; gel B). *In situ* tryptic digestions of the selected spots excised from the two-dimensional gels, followed by MALDI–TOF–TOF peptide mass fingerprinting, identified 152 different proteins. Thus, we were able to detect about 10% of the complete BAA-1163 predicted proteome. Several isoforms were found probably due to post-translational modifications or to artefacts due to sample handling. In gel B, 12 different proteins were identified among the 21 spots characterized; seven of these were also detected in the total protein fractions (gel A). The list of identified proteins and their physico-chemical properties as well as the parameters used for their identification are presented in the electronic supplementary material, table S1.

### 4.2. Physico-chemical analysis of the identified proteins

**Figure 2.**
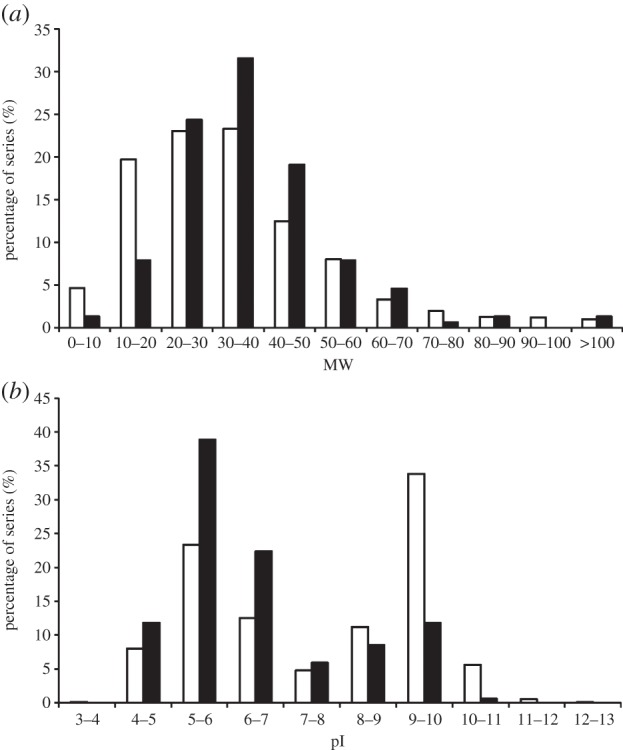
Comparative analyses of the predicted and the actual proteome of *O. oeni* ATCC BAA-1163. Frequency distribution of the MW (*a*) and pI (*b*) of the whole set of putative proteins encoded by the genome (white bars) and of the 152 proteins identified in this work (black bars).

The ATCC BAA-1163 proteome map reported here contains 152 proteins, mainly characterized by a MW lower than 50 kDa (84%; figure 2*a*) or acidic pI, with approximately 40% of the identified peptides within the range of pI 5.0–6.0 (figure 2*b*). Only 41 proteins possessed an alkaline pI (the electronic supplementary material, table S1; figure 2*b*). This contrasts with the theoretical prediction that 55% of the BAA-1163 proteome consists of proteins that have a pI within the range of 9.0–10.0 (figure 2*b*). This discrepancy is probably an artefact owing to the methodology. Specific reference maps for the alkaline proteome have been reported for *Lactococcus lactis* [27] and *Lactobacillus acidophilus* [28]. Therefore, this region of the BAA-1163 proteome should be further investigated.

**Figure 3.**
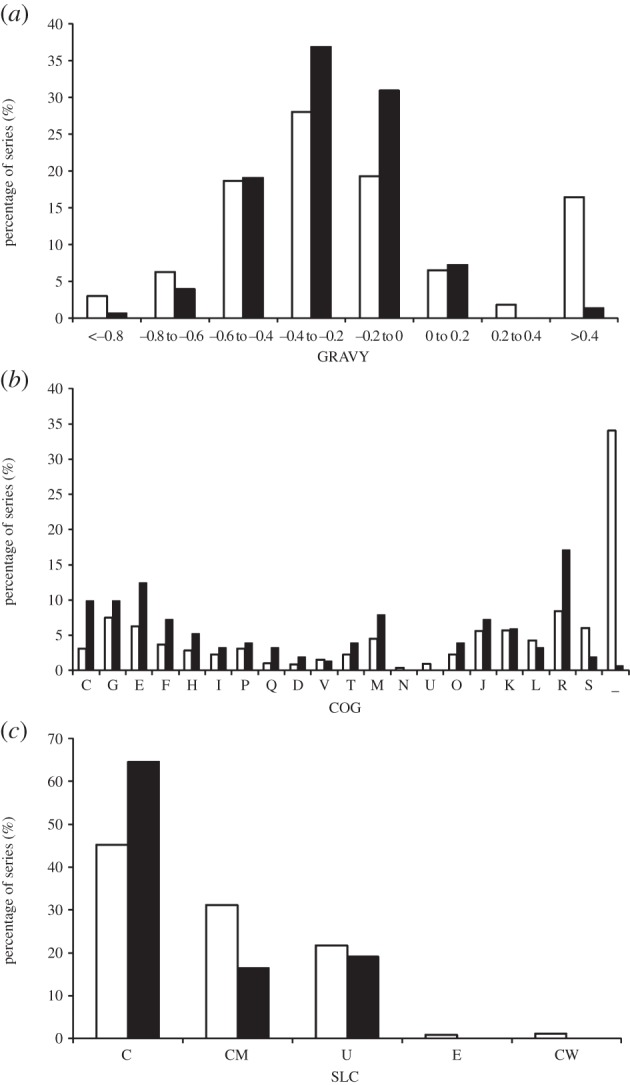
Comparative analyses of the predicted and the actual proteome of *O. oeni* ATCC BAA-1163. Frequency distribution of the GRAVY index (*a*), COG categories (*b*) and subcellular location (*c*) of the theoretical proteome (white bars) and of the identified proteins (black bars).

The grand average of hydrophobicity (GRAVY) index was calculated by using the GRAVY calculator tool (http://www.gravy-calculator.de) for the identified proteins. According to previous reported proteomes from *Bifidobacterium longum* and different LAB [27–31], most of the identified proteins (more than 90% of the total) showed a negative GRAVY index (figure 3*a*), confirming the difficulty of separating hydrophobic proteins on two-dimensional gels. The most hydrophilic protein found in this work was the heat-shock protein Lo18 (−0.941). A set of 13 proteins with a GRAVY index of 0.0 or higher were detected, five of these were separated on gel B (the electronic supplementary material, table S1). In particular, the highest hydrophobic index was observed for a phosphate ABC transporter permease subunit showing a GRAVY value of 0.752. Concordantly, this subunit has six transmembrane domains predicted by the TMpred program (data not shown).

Experimentally identified proteins were grouped into cellular roles according to COG (Clusters of Orthologous Groups) categories on the basis of biological or biochemical function (figure 3*b*). The most abundant group contained proteins involved in metabolism (55% of the total), especially those involved in the metabolism of amino acids (E) and the pathways related to energy production and conversion (C). Other abundant categories included proteins implicated in cell wall/membrane biogenesis (M) and related to translation (J). Subcellular localizations (SLC) of all 152 identified proteins, predicted by PSORT v. 3.0 [32] (figure 3*c*), showed 98 cytoplasmic proteins (65% of the total identified proteins), 25 proteins (16%) predicted to be located in the cytoplasmic membrane and 29 proteins (19%) of unknown cellular locality. Our results correlate with the reported proteome of *B. longum* NCC2705 [29], in which from a total of 369 identified proteins, only 5.3 and 9.3% were predicted to be located at the cytoplasmic membrane or in an unknown location, respectively. Likewise, in the proteomes of *Lactobacillus plantarum* CMCC-P0002 [31] and *Lactobacillus acidophilus* NCFM [28], the authors reported that less than 10% of the identified proteins are predicted to be present in the cytoplasmic membrane. Our results are more similar to those observed by Choi *et al*. [25], who found approximately 18% of membrane-associated proteins in extracts of *S. pneumoniae*. Moreover, these authors identified only two cell wall proteins in fractions obtained by ultracentrifugation and treatment with ammonium carbonate, which accords with our two-dimensional gel analysis where we were unable to detect the predicted 16 cell wall proteins of *O. oeni*, representing 1% of the theoretical proteome. Six proteins were predicted by the SignalP program to have a signal peptide, namely the hypothetical protein OENOO_49004, the substrate-binding component (OENOO_31002) of an ABC oligopeptide transport system, the permease component PstD (OENOO_58021) of a phosphate transport system, serine-type d-Ala-d-Ala carboxypeptidase OENOO_60073, located at the cytoplasmic membrane level as well as the UDP-glucose 6-dehydrogenase OENOO_64076 (with unknown subcellular location) and the UTP-glucose-1-phosphate uridylyltransferase OENOO_58029 (cytoplasmic location).

### 4.3. Hypothetical proteins

The DNA sequence of *O. oeni* ATCC BAA-1163 genome is still in a provisional state of annotation. Nevertheless, all the proteins described in this study were identified by reference to this genome.

Proteomic analysis is a powerful tool to prove the existence of hypothetical gene products. In this work, we confirmed the occurrence of 14 proteins previously designated as hypothetical that should now be considered as real proteins (the electronic supplementary material, table S2). A BLAST search of these proteins against the Conserved Domain Database (http://www.ncbi.nlm.nih.gov/Structure/cdd/cdd.shtml) and similarity to homologues predicted a possible function for 10 hypothetical proteins based on the conserved domains (the electronic supplementary material, table S2). SMART biotools (http://smart.embl-heidelberg.de/) was used for the motif search. In particular, we found that the hypothetical protein OENOO_53026 could be a secreted small protein. This prediction is supported by its unknown SLC and the occurrence of a transmembrane domain. Extra-cytoplasmic proteins often have roles in establishing and maintaining interactions between a bacterium and its environment. In *O. oeni*, secreted proteins have been reported for their proteolytic activity, crucial for survival in the wine environment [33]. One hypothetical protein, OENOO_37002, possesses the domain characteristics of the universal stress protein UspA, suggesting its involvement in stress response. UspA is highly expressed in response to heat, starvation, exposure to antimicrobial agents and oxidative stress [34,35]. Recently, Gury *et al*. [36] demonstrated that in *L. plantarum* NC8 the putative universal stress protein Usp1 is involved in phenolic acid stress response. The GRAVY index of the hypothetical stress protein (−0.018) indicated the presence of hydrophobic regions suggesting their interaction with the membrane. The cytoplasmic membrane is an important barrier with the external environment and stress proteins can be associated with cellular membrane fractions [15]. The role of the small heat-shock protein Lo18 from *O. oeni* in the modulation of membrane fluidity has been reported recently [37] and the protein from *O. oeni* ATCC BAA-1163 (OENOO_66120) has been identified in this work (the electronic supplementary material, table S1).

A set of three proteins characterized by the domains Metallophos (OENOO_06001 and OENOO_47010) and PRTases_type I (OENOO_66092) with phosphoesterase functions are possibly involved in nucleic acid biosynthesis and repair. Hypothetical protein OENOO_54035 contains a Glo_EDI_BRP-like domain, which is a characteristic region in enzymes that degrade aromatic compounds. Hypothetical protein OENOO_45020 carries the ACT domain characteristic of metabolic enzymes regulated by the amino acid concentration. Also, the conserved DUF4230 domain of unknown function was detected in OENOO_49004. Finally, the hypothetical protein OENOO_63029 seems to be implicated in the pathway for peptidoglycan biosynthesis. No domains were identified for the conserved hypothetical protein (OENOO_38016), the site-specific DNA-methyltransferase (OENOO_53044) and the hypothetical proteins OENOO_63062, OENOO_6403 and OENOO_6606. The latter possesses three transmembrane domains and presumably is an integral membrane protein.

### 4.4. *Oenococcus oeni* ATTC BAA-1163 strain-specific proteins

Recently, a comparative analysis has been carried out on all of the 14 available *O. oeni* genomes [5]. The *in silico* analysis revealed 2846 non-degenerate ORFs that were shown to comprise the chromosomal pan genome of *O. oeni*, with 1165 of these being core ORFs conserved across all the strains [5,38]. Accordingly, we investigated the occurrence of the polypeptides identified in ATCC BAA-1163 within the genomes of PSU-1 and the 12 sequenced AWRIB strains. Based on the inferred protein sequence homology, we found that genes encoding 14 proteins were present in the genome of at least one of the considered strains, but not reported as encoding proteins probably owing to an incorrect annotation (the electronic supplementary material, table S3).

The genes encoding two proteins (oxidoreductase OENOO_40005 and hypothetical protein OENOO_63029) were absent in the genomes of all of the examined strains. Furthermore, oxidoreductase OENOO_40005, a functionally predicted nucleoside-diphosphate-sugar epimerase (the electronic supplementary material, table S1), had a 99% of identity with its predicted homologues in *Lactobacillus vini* and *Lactobacillus casei*. Strain-specific genes of *O. oeni* with an increased probability of being horizontally acquired from *Lactobacillus* spp. have been investigated *in silico* [5]. The analysis of the surrounding region of OENOO_40005 revealed a downstream gene encoding for a phosphotransferase system (PTS) cellobiose-specific component IIC, supporting the existence of strain-dependent genomic insertion events correlated with differences in carbohydrate utilization [5]. In this work, we found that the hypothetical protein OENOO_63029 belonging to the pyruvyl-transferases family (the electronic supplementary material, table S2) involved in peptidoglycan-associated polymer biosynthesis was expressed in FT80 (figure 1), a model semi-defined medium for growth of *O. oeni* containing glucose and fructose as carbon sources. The hypothetical protein OENOO_63029 coding gene is located between the loci (EAV38986 and EAV39008) encoding the aminotransferase OENOO_63016 and the PTS system, sucrose-specific IIBC component OENOO_63040 in ATCC BAA-1163. Neither this gene nor the corresponding locus is present in the genome of the other 13 sequenced strains. This region contains genes encoding for exopolysaccharides (EPS) biosynthesis and different PTS systems, including a PTS for fructose-specific utilization. However, in *O. oeni* differences in EPS production across the strains have been associated with variation in the EPS loci [5,38]. The strain-variable ability of *O. oeni* to synthesize EPS has been investigated for its technological and biological interest [39–41]. ATCC BAA-1163 was found to be a non-ropy strain able to produce low amounts of EPS in an MRS-derived media [40]. The same strain produces significantly more EPS in a semi-defined medium supplemented with a mixture of glucose and sucrose [41].

With regard to esterase C (OENOO_51026), the protein-coding gene is absent in the genomes of the AWRIB422 and AWRIB548 strains and it is truncated prior to the active site-coding region in all the other strains, apart from AWRIB419 (the electronic supplementary material, table S3). This frequency of non-functional genes correlates with a previous study performed by Sumby *et al*. [42]. The authors reported that sequencing of the *EstC* of 20 *O. oeni* strains, whose genomes have not been characterized, revealed that in only four strains the gene was not carrying an early stop codon. Moreover, the analysis of ATCC BAA-1163, PSU-1 and AWRIB429 revealed that only the former carries an entire *EstC* gene [42]. The significance of the presence of truncated genes in most of the strains is unclear but suggests that the gene tends to be lost during evolution. This could be owing to the existence of other esterases in *O. oeni* like EstA, which we have not identified in this work, that could eliminate the requirement for EstC. Sumby *et al*. [42] proposed that it should be investigated whether EstC has an effect on strain-specific differences in ester hydrolysis and synthesis in wine.

### 4.5. Overview of metabolic pathways

**Figure 4.**
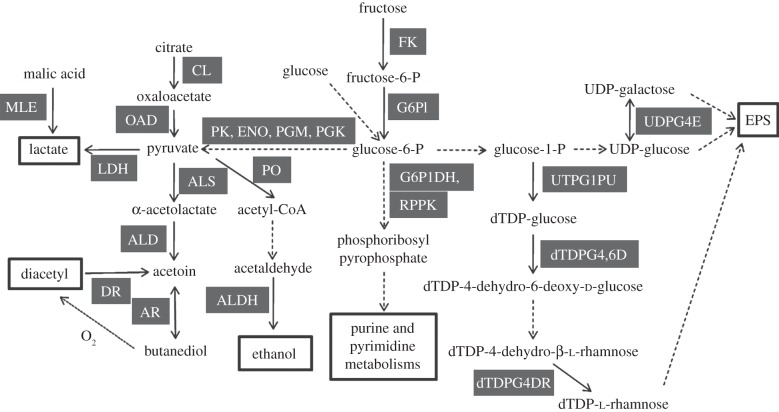
Proteomic and genomic prediction of metabolic pathways in *O. oeni* ATCC BAA-1163. Enzymatic reactions: continuous lines indicate proteomic detection of the catalysing enzyme; dashed arrow lines indicate detection of the enzyme-coding gene in the bacterial genome. MLE, malolactic enzyme; LDH, lactate dehydrogenase; CL, citrate lyase; OAD, oxaloacetate decarboxylase; ALS, α-acetolactate synthase; ALD, α-acetolactate decarboxylase; AR, acetoin reductase; DR, diacetyl reductase; PGK, phosphoglycerate kinase; PGM, phosphoglycerate mutase; ENO, enolase; PK, pyruvate kinase; PO, pyruvate oxidase; ALDH, alcohol dehydrogenase; FK, fructokinase; G6PI, glucose-6-phosphate isomerase; G6P1DH, glucose-6-phosphate 1-dehydrogenase; RPPK, ribose-phosphate pyrophosphokinase; UTPG1PU, UTP-glucose-1-phosphate uridylyltransferase; dTDPG4,6D, dTDP glucose 4,6-dehydratase; dTDPG4DR, dTDP-4-dehydrorhamnose reductase; UDPG4E, UDP-glucose 4-epimerase.

A scheme for the most significant reconstructed metabolic pathways based in the proteomic data (figure 1 and the electronic supplementary material, table S1) and supported by the analysis of *O. oeni* ATCC BAA-1163 genome is illustrated in figure 4. *Oenococcus oeni* is a heterofermentative microorganism able to use hexoses and pentoses *via* the phosphoketolase pathway [43]. Expression of enzymes of central metabolism, such as the glycolytic (four out of 10 proteins), pentose phosphate and pyruvate biosynthetic, and metabolic (including ethanol production) pathways are reported in the present map (figure 4). Also enzymes involved in citrate and malic acid utilization were identified (figure 4). In addition, we detected UTP-glucose-1-phosphate uridylyltransferase (OENOO_58029), UDP-glucose 6-dehydrogenase (OENOO_64076) and UDP-glucose 4-epimerase (OENOO_50027), which are involved in several pathways of carbohydrate metabolism, such as pentose and glucuronate interconversions, galactose, ascorbate and aldarate, starch and sucrose, amino sugar and nucleotide sugar metabolism. ATCC BAA-1163 has been described as being able to use l-arabinose, arabinan, sucrose and d-sorbitol through their conversion to d-fructose [5]. However, in this work we were unable to detect the corresponding enzymes.

**Figure 5.**
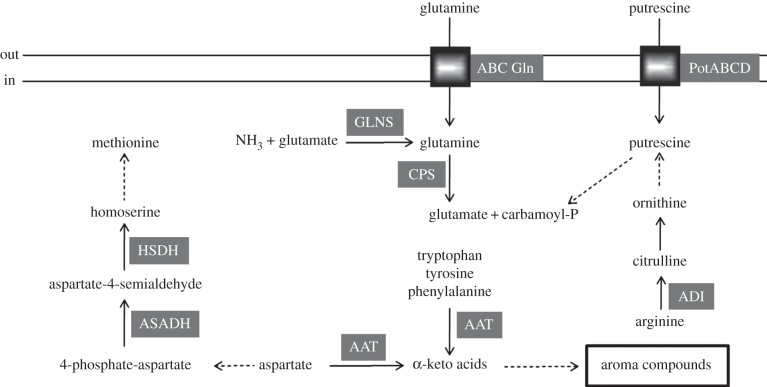
Proteomic and genomic prediction of amino acid metabolism in *O. oeni* ATCC BAA-1163. Enzymatic reactions: continuous arrow lines indicate proteomic detection of the catalysing enzyme; dashed lines indicate detection of the enzyme-coding gene in the bacterial genome. ABC Gln, glutamine ABC transporter; PotABCD, putrescine ABC transporter; GLNS, glutamine synthetase; CPS, carbamoyl-phosphate synthase; ADI, arginine deiminase; AAT, arginine/aromatic amino acids aminotransferase; ASADH, aspartate-semialdehyde dehydrogenase; HSDH, homoserine dehydrogenase.

Previous analyses of *O. oeni* strains have revealed a large number of auxotrophies [44,45]. In particular, the requirement for amino acids seems to be related to a genomic intra-species diversity [4,5]. We found enzymes involved in several amino acid metabolic pathways, including methionine biosynthesis from aspartate, glutamine utilization and conversion in glutamic acid and arginine as well as aromatic amino acids catabolism (figure 5). However, we were unable to detect proteins for phenylalanine, tyrosine and tryptophan biosynthesis, nor could we detect the coding genes in the ATCC BAA-1163 genome. In agreement with these findings, Remize *et al*. [45] showed that phenylalanine and tyrosine were essential for growth of the strain in FT80 media and they detected only a residual growth (20% of the control) in the absence of tryptophan supporting the predicted amino acids auxotrophies.

After the alcoholic fermentation, the free amino acid concentration in the must increases owing to the autolysis of the yeasts, with arginine usually being the most abundant. In our proteomic map (figure 1 and the electronic supplementary material, table S1), we found the OENOO_57007 arginine deiminase (ADI), a key enzyme in arginine catabolism ([46] and figure 5). Analysis of the bacterial genome confirmed that ATCC BAA-1163 possesses the *arcABC* operon encoding the three enzymes that constitute the ADI pathway involved in counteracting stress and associated with the potential production of the toxic compound putrescine (figure 5). Putrescine is one of the most commonly occurring biogenic amines in wine [47] and can be taken up into the cell *via* the ABC transporter complex PotABCD, responsible for energy coupling to the transport system. As we detected PotA, the ATP-binding subunit (OENOO_19003 in the electronic supplementary material, table S1) of the spermidine/putrescine transporter during the growth of ATCC BAA-1163 in a synthetic medium, it could be argued that the intake of polyamine is not purely a mechanism to cope with the harsh wine environment. The importance of exchanging a wide variety of substrates is supported by the transporters identified in this work, namely two ABC-type oligopeptide transport systems, three subunits of one (OENOO_31002, OENOO_30004 and OENOO_30005) and the ATP-binding cassette (OENOO_31006) of the other and also the ATP-binding proteins of a glutamine ABC transporter (OENOO_41008), a multiple sugar ABC transporter (OENOO_37007) and an uncharacterized ABC transporter (OENOO_65021). In addition, we identified subunits of the ABC transporters associated with iron (OENOO_57011 and OENOO_57012), cobalt (OENOO_44010) and phosphate (OENOO_58021, OENOO_58023 and OENOO_58024) metabolism.

We detected three proteins, namely UTP-glucose-1-phosphate uridylyltransferase (OENOO_59028), dTDP glucose 4,6-dehydratase (OENOO_59030) and dTDP-4-dehydrorhamnose reductase (OENOO_59032), whose coding genes are probably organized into an operon, and being part of the nucleotide sugars biosynthetic pathway (figure 4) seem to provide the substrates for the synthesis of heteropolysaccharides catalysed by glycosyltransferases, whose coding genes are located in the specific region OENOO_63027 and OENOO_63040 in the ATCC BAA-1163 genome.

In recent years, several works have investigated the contribution of the malolactic fermentation and of the strain-specific variability of *O. oeni* on the modulation of the wine's flavour profile [10,48,49]. For example, it is well known that compounds such as diacetyl, acetoin and 2,3-butanediol from citric acid metabolism via pyruvate can affect the aromatic complexity of wine [50]. These molecules responsible for buttery and nutty fragrances proceed from pyruvate by the activity of the enzymes α-acetolactate synthase (OENOO_54033), α-acetolactate decarboxylase (OENOO_54034), acetoin reductase (OENOO_48023) and diacetyl reductase (OENOO_43013). All these proteins were identified in our two-dimensional electrophoresis analysis (figures 1 and 4; the electronic supplementary material, table S1). Moreover, the enzymes involved in conversion of citrate to pyruvate—two subunits (α and β, OENOO_66031 and OENOO_66032) of the citrate lyase, CitXG protein, which includes Apo-citrate lyase phosphoribosyl-dephospho-CoA transferase (OENOO_66029) and the oxaloacetate decarboxylase (OENOO_66036)—were also identified and their coding genes are located in a cluster that also includes the determinant of the putative citrate transporter MaeP [51]. Furthermore, we identified two putative aspartate/aromatic amino acid aminotransferases (OENOO_47017 and OENOO_60020). Transamination reactions have recently attracted attention, because they are the first step for the synthesis of important flavour or aroma compounds in amino acid catabolism pathways. Finally, esters from microbial metabolism often underlie the fruit aroma of the wine [38,52]. Some authors have suggested the use of purified esterases from LAB, including *O. oeni*, as additives in winemaking [53,54]. For this reason, the identified protein (the electronic supplementary material, table S1) tributyrin esterase (OENOO_60072), and in particular, the esterase C (OENOO_51026), which does not belong to the core genome of *O. oeni*, deserves to be the focus of further investigation. Thus, recently the esterase C from *O. oeni* has been overproduced in *Escherichia coli* and biochemical characterization of the purified enzyme under conditions relevant to winemaking indicate that indeed esterase C is a potential candidate to alter the ester profile of wine [42].

## 5. Concluding remarks

Using standardized extraction techniques and two-dimensional gel electrophoresis coupled with MS/MS analysis, we identified 224 different spot polypeptides, corresponding to 152 unique proteins, from *O. oeni* ATCC BAA-1163. This represents approximately 10% of the BAA-1163 predicted proteome. A total of 21 spots were associated with the membrane preparation. The methodology was found to cause some bias in the types of proteins identified, e.g. proteins in the MW range of 30–50 kDa were over-represented compared with the predicted proteome, as were proteins in the pI range 5–6 (contrasting with the prediction that 55% of BAA-1163 proteins have a pI within the range of 9–10). Hydrophobic and very hydrophilic proteins were under-represented.

The analysis allowed the detection of a wide variety of metabolic enzymes, including many involved in the synthesis and catabolism of various carbohydrates, amino acids and amines. This study should, therefore, be helpful to those researching the biochemistry of *O. oeni* ATCC BAA-1163.

## Supplementary Material

Table S1

## Supplementary Material

Table S2

## Supplementary Material

Table S3

## Data accessibility

The mass spectrometry proteomics data have been deposited in the ProteomeXchange Consortium (http://proteomecentral.proteomexchange.org) via the PRIDE partner repository [55] with the dataset identifier PXD000579 and doi: 10.6019/PXD000579.
